# The role of peroxisome proliferator-activated receptors in endometriosis

**DOI:** 10.3389/fmed.2024.1329406

**Published:** 2024-04-16

**Authors:** Iason Psilopatis, Stamatios Theocharis, Matthias W. Beckmann

**Affiliations:** ^1^Department of Gynecology and Obstetrics, Erlangen University Hospital, Friedrich Alexander University of Erlangen-Nuremberg, Comprehensive Cancer Center Erlangen-EMN, Erlangen, Germany; ^2^First Department of Pathology, Medical School, National and Kapodistrian University of Athens, Athens, Greece

**Keywords:** endometriosis, peroxisome proliferator-activated receptors, epigenetics, inflammation, vascularization

## Abstract

Endometriosis constitutes the most common cause of chronic pelvic pain in female patients and is associated with infertility. Although there is no known cause for the disease, it is a heritable condition that is determined by numerous genetic, epigenetic, and environmental aspects. Peroxisome proliferator-activated receptors (PPARs) represent nuclear receptor proteins that control gene expression. By using the MEDLINE and LIVIVO databases we conducted a literature review in order to look into the role of PPARs in the endometriosis pathophysiology and succeeded in revealing 36 pertinent publications between 2001 and 2022. In regards to PPAR expression in endometriosis, PPARγ seems to represent the most studied PPAR isoform in endometriosis and to influence various pathways involved in the disease onset and progression. It's interesting to note that diverse treatment agents targeting the PPAR system have been identified as innovative, effective therapeutic alternatives in the context of endometriosis treatment. In conclusion, PPARs appear to contribute an important role in both endometriosis pathophysiology and therapy.

## 1 Introduction

Endometrial tissue growing outside of the womb in females of reproductive age is known as endometriosis, a chronic disease (1–3). Depending on the localization of the different endometriotic lesions, various clinical characteristics of endometriosis exist (4, 5). More precisely, patients with endometriosis typically report dyspareunia, chronic pelvic pain, alongside with dysmenorrhea, vaginal bleeding and/or infertility (6). Affected patients may continue to be asymptomatic. Should endometriotic lesions invade the urinary tract, dys-/hematuria can occur, while dys-/hematochezia represent additional symptoms upon intestinal involvement (7). Initial diagnosis of endometriosis is mostly based on transvaginal ultrasound findings, in addition to a thorough physical examination and patient history (8, 9). The uterus is typically not engorged, but the existence of ovarian cysts or nodules in the rectovaginal septum and/or the bladder necessitates additional examinations (10). The best confirmatory test currently available is considered to be laparoscopy because it can detect endometriotic adhesions and implants (11). Most asymptomatic women should receive expectant management (12). When there are no complications and moderate pelvic pain, the use of analgesics in combination with continuous hormonal contraceptives is appropriate for treating symptomatic endometriosis (13). Gonadotropin-Releasing Hormone (GnRH) agonists or oral estrogen-progestin contraceptives may need to be administered if symptoms are severe (14, 15). Laparoscopic excision and thermal destruction of endometrial lesions are the most widely spread surgical treatments when pharmacological therapy fails to work or the disease course is complicated (16). Both retrograde menstruation and coelomic metaplasia are the two most common pathogenetic hypotheses, but the exact pathophysiology of endometriosis development and progression are still not fully explored (17, 18). The hematogenous/lymphatic spread theory, the embryogenetic theory, and the stem cell recruitment theory are additional pertinent hypotheses (17).

Nuclear receptors which get activated by fatty acids are known as peroxisome proliferator-activated receptors (PPARs) (19). The three PPAR isoforms PPARα, PPARβ/δ, and PPARγ, each of which has distinct metabolic regulatory functions, tissue distribution, and ligand-binding characteristics (20, 21), are all present in the body. PPARs may form heterodimers with the retinoid X receptors (RXRs) and bind to particular DNA response elements within promoter regions hence effectively inducing or repressing the expression of their target genes (22). Given the larger PPAR ligand binding cavity's size, diverse natural and synthetic ligands may attach, causing the replacement of co-repressors from co-activators and thereby enhancing the roles of PPARs (23, 24). Only a few of the processes that PPARs control include fatty acid disposition and metabolism, various cellular biology processes, energy homeostasis, cell differentiation and immunity mechanisms (25, 26). More specifically, PPARα co-determines fatty acid metabolism and is highly expressed in several organs such as the heart or the skeletal muscles, liver, intestinal tract, kidneys, and brown adipose tissue. Fatty acid oxidation is regulated by PPARβ/δ, which is widely expressed and also regulates blood sugar and cholesterol levels. When it comes to lipoprotein metabolism lipid biosynthesis, adipogenesis, and insulin sensitivity, PPARγ is most highly expressed in adipose cells (23, 27).

**Figure 1:**
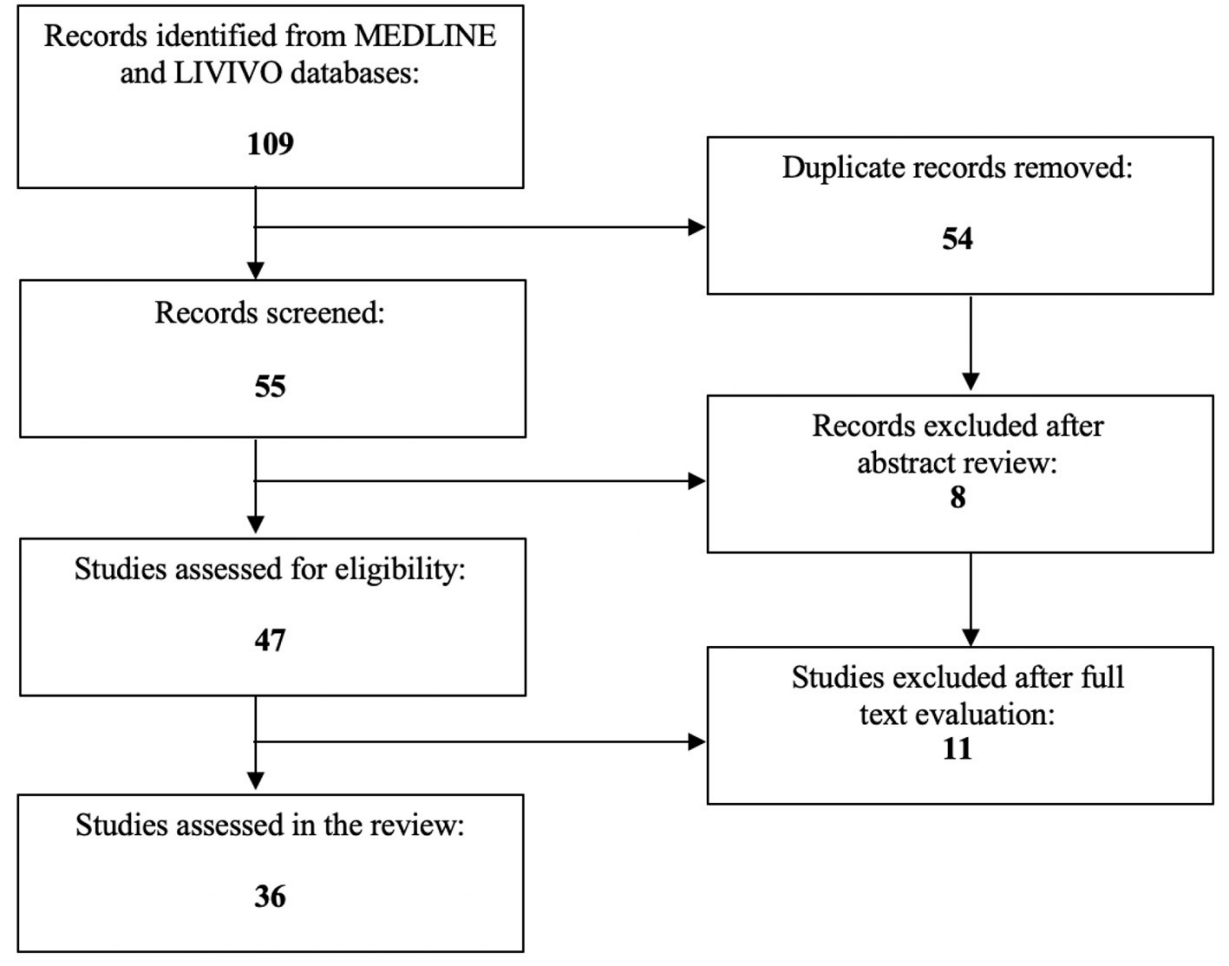
PRISMA flow diagram visually summarizing the screening process.

To date, PPARs have been proclaimed to directly or indirectly co-determine numerous procedures associated with cancer and/or gynecological health conditions (28–31). The focus of the present literature review is to investigate the exact role of PPARs in endometriosis. The MEDLINE and LIVIVO databases were used for the conduction of the literature search. In order to be eligible for inclusion, manuscripts needed to embody original research articles and scientific abstracts written in the English language that clearly reported on the role of PPARs in this gynecologic disease. Studies on the involvement of PPARs in uterine fibroids or endometrial cancer had to be excluded. The search terms “peroxisome proliferator-activated receptor,” “PPAR,” and “endometriosis” were employed. After the exclusion of duplicates a total of 55 articles published between 2001 and 2023 were identified. In the initial selection process, a total of eight works had to be discarded after abstract review. After detailed analysis of the full texts of the remaining 47 publications, a total of 36 relevant studies published between 2001 and 2022, that met the inclusion criteria, could be selected for the literature review. Figure 1 depicts the aforementioned selection process.

## 2 The role of genetics-epigenetics in endometriosis

According to the genetic-epigenetic (G-E) theory, endometriosis doesn't actually start until a number of cumulative G-E cellular changes have taken place. Predisposed women with more inherited incidents are hence at higher risk, while endometriosis could develop from any pluripotent cell other than the endometrium. This theory also explains why “endometrium-like cells” can harbor significant G-E differences. In this context, endometriosis cannot begin to develop until a number of cumulative G-E incidents have occurred (32). Errors can occur during cell division, and the risk is likely to rise in the presence of dioxin, oxidative stress, or ionizing radiation. The endometrium has a higher risk because it is the tissue in our body that grows the fastest. In addition, retrograde menstruation and microbiota, alongside the oxidative stress of the peritoneal cavity, are specifically associated with endometriosis (33, 34). In endometriosis, the frequent cancer-driver mutations brought on by genomic instability are examples of DNA mutations (35–37). Epigenetic incidents in particular have not yet been recorded. Nonetheless, one challenge is that epigenetic reorganizations can be too complex or even irreversible. Therefore, despite an eventual similarity in histology, the G-E theory proposes that the endometriotic cell has experienced irreversible G and/or E changes, permanently separating endometriosis from the endometrium (32). The possibility that these endometriotic cells could cause reversible metaplasia in the surrounding cells, which would cause them to histologically resemble endometrium, is currently more speculative. This is strongly implied, though not formally proven, when it is remembered that recurrences rise even after resections with safety margins. According to the G-E theory, endometriosis can develop from any type of poorly differentiated cell such as stem and bone marrow cells. Nonetheless, there is a higher chance of development from endometrium or embryological rests that have already progressed in that way (32). Each endometriosis lesion has a unique number and type of G-E incidents because they are clonal and have different molecular biology, which explains the various levels of progesterone resistance or aromatase activity (38, 39). This variation also provides an explanation why some lesions are not painful or do not cause distant discomfort (40) and why different patients may respond differently to hormonal therapy (41, 42). The specific arrangement of G-E incidents may also account for the emergence of superficial, cystic, or deep endometriosis in lesions. It is also crucial to note that the numerous endometriosis-related changes in the endometrium, immunology, infertility, and pregnancy are not always caused by endometriosis but can instead be attributed to an inherited propensity (32). Deep endometriosis in women not taking estrogens can occasionally start and progress years after menopause, which is also explained by the G-E pathogenesis (32).

## 3 The role of PPARs in the pathogenesis of endometriosis

Chen et al. performed a Gene Ontology and Kyoto Encyclopedia of Genes and Genomes (KEGG) pathway analysis and stated that the PPAR signaling pathway is associated with the proliferation, metastasis, and autophagy of endometrial cells (43). Moreover, Hornung et al. evaluated the role of PPARs in macrophage attraction into the peritoneal cavity of women with endometriotic lesions. Using immunocytochemistry, the researchers localized PPARα and PPARγ within the nuclei of peritoneal macrophages and pro-monocytic, human histiocytic lymphoma U937 cells. Migration of U937 cells was increased by the PPARα agonist WY14643 and restrained by rosiglitazone. Peritoneal fluid from patients with endometriosis activated U937 cells which had been transiently transfected with a PPARα reporter, whereas it did not successfully activate PPARγ constructs. Notably, U937 cells, which had been transiently transfected with a PPAR reporter, showed endometriosis stage-dependent up-regulation upon peritoneal fluid treatment from women with endometriosis. On the contrary, PPAR response element transactivation was down-regulated after treatment with peritoneal fluid from healthy controls (44).

### 3.1 The role of PPARα in the pathogenesis of endometriosis

Two studies have, so far, examined the role of PPARα in endometriosis.

Peng et al. (45) demonstrated that high local estrogen level derived from ectopic endometrial stromal cells promote expression of prion, which serves as a critical mediator in augmenting cholesterol accumulation and estrogen production through negative regulation of the PPARα pathway, thus promoting endometriosis progression. Furthermore, Pergaliotis et al. examined mice with surgically-induced endometriosis and reported that PPARα-deficient mice cannot properly induce angiogenesis to sustain as many endometriotic crypts in the implantation site, with the amount of inflammation in the implantation site hence being significantly higher compared to control animal. However, in the control group, fibroblastic activity was found to be significantly up-regulated (46).

### 3.2 The therapeutic effects of PPARα-targeting agents in endometriosis

Chen et al. generated a first model of endometriosis in rats and a second model of endometriosis using the human ectopic endometrial stromal cells (HEcESCs) derived from the lesion tissues of endometriosis patients. Following the administration of resveratrol in the rat model, significant efficacy was observed in the context of lesion size attenuation and aberrant lipid profile rectification. Lipidomic analysis revealed, on the one hand, notable sphingolipid increases and, on the other hand, decreases of both glycerolipids and most phospholipids. Upon resveratrol admission, both proliferation capacity and invasiveness parameters diminished, whereas the early apoptosis proportion augmented for HEcESCs. The activation of PPARα was also described as a potential factor of recovery from endometriosis in both models (47). Massimi et al. (48) treated 12Z endometriotic epithelial cells with aspirin and other non-aspirin Non-Steroidal Anti-Inflammatory Drugs (NSAIDs) which induced PPARα expression.

### 3.3 The role of PPARγ in the pathogenesis of endometriosis

The most studied PPAR in endometriosis is undoubtedly PPARγ.

Caserta et al. (49) suggested that patients with endometriosis show higher PPARγ levels than women with other causes of infertility. Additionally, Liu et al. found that both ovarian endometriomas and deep infiltrating endometriosis had significantly reduced immunoreactivity against PPARγ. In particular, PPARγ staining levels were significantly lower in the deep infiltrating endometriosis patient group. Of note, the PPARγ staining levels was negatively associated with the fibrotic extent (50). Besides, Zolbin et al. performed a gene expression analysis and showed that endometriosis may alter the expression of PPARγ. More precisely, the let-7b mimic significantly reduced the messenger RiboNucleic Acid (mRNA) levels of PPARγ, whereas microRNA-342-3p mimic increased its expression levels (51). Harzif et al. presented the case of a 34-year-old patient with extrapelvic endometriosis in an abdominal wall scar. A sample of the abdominal endometriosis and uterine endometrium underwent Reverse Transcriptase quantitative Polymerase Chain Reaction (RT-qPCR) examination of PPARγ mRNA expression. PCR examination results revealed that PPARγ activity was higher in the abdominal wall endometriosis compared to the eutopic endometrium (52).

Dogan et al. investigated the correlation of the PPAR-γ2 Proline to Alanine at 12th amino acid (Pro12Ala) polymorphism with endometriosis in a case–control study incorporating 55 control women without endometriosis as opposed to 51 women with endometriosis stages I–IV. The study group observed a higher frequency of the Pro-12-Ala polymorphism in women with endometriosis compared with the control cases. Interestingly, the frequency was even greater in patients with one or more recurrences of endometriosis, thus implying that the 12-Pro allele might exert protective effects in terms of implantation and growth of ectopic endometrial fragments, whereas the 12-Ala allele could potentially facilitate endometrioid development, progression, and recurrence (53). Moreover, Kiyomizu et al. proved that there is no association between distribution of genotype or allele frequencies for the PPAR-γ Pro12Ala polymorphism and the presence of adenomyosis and/or endometriosis in the Japanese population. Nonetheless, the PPAR-γ 161CC genotype and 161C allele frequencies seem to show significant overexpression in women with adenomyosis and/or endometriosis (54). In a similar context, Hwang et al. performed a case–control study in a collective of 427 controls and 446 patients in the Korean population. The distribution of the PPAR-γ2 Pro12Ala polymorphism differed between the advanced-stage endometriosis group and the control group, while the frequency for the Ala-12 allele variant was significantly higher in the control group than in patients with advanced endometriosis stages (55). Last but not least, Beeram et al. reported that genetic variants of the PPARγ coactivator 1α (PGC-1α) do not seem to correlate with the risk of endometriosis in the Indian population, with GTT of PGC-1α gene constituting the most common haplotype in Indian women (56).

Table 1 summarizes the role of PPARγ in the pathogenesis of endometriosis.

**Table 1:** The role of PPARγ in the pathogenesis of endometriosis.

**Study**	**Endometriosis entity**	**Main results**
Caserta et al. (49)	Endometriosis (not nearly specified)	Endometriosis patients show higher levels of PPARγ than women with other causes for infertility
Liu et al. (50)	Ovarian endometrioma Deep infiltrating endometriosis	Significantly reduced immunoreactivity against PPARγ
Zolbin et al. (51)	Endometriosis (not nearly specified)	Let-7b mimic significantly reduces PPARγ mRNA levels microRNA-342-3p mimic increases PPARγ mRNA levels
Harzif et al. (52)	Extrapelvic endometriosis in an abdominal wall scar	PPARγ activity is higher in abdominal wall endometriosis compared to eutopic endometrium
Dogan et al. (53)	Endometriosis stages I–IV	Higher frequency of the Pro-12-Ala polymorphism in patients with endometriosis
Kiyomizu et al. (54)	Adenomyosis Endometriosis (not nearly specified)	No association between distribution of genotype or allele frequencies for the PPAR-γ Pro12Ala polymorphism and the presence of adenomyosis and/or endometriosis in the Japanese population
Hwang et al. (55)	Advanced-stage endometriosis	PPAR-γ2 Pro12Ala polymorphism frequency for the Ala-12 allele variant is significantly lower in Korean patients with advanced stage of endometriosis
Beeram et al. (56)	Endometriosis (not nearly specified)	Genetic variants of PGC-1α does not correlate with the endometriosis risk in the Indian population

### 3.4 The PPARγ-associated therapeutic effects of antihypertensive, antidiabetic and cholesterol-regulating agents in endometriosis

Glitazones represent synthetic derivatives of thiazolidinedione, and are designated as potent oral anti-diabetic agents (57). A Turkish study group transplanted endometrial tissue fragments onto the inner surface of the abdominal wall in 28 rats and, consecutively, orally administered rosiglitazone to this group. Surprisingly enough, the PPARγ agonist succeeded in significantly reducing the height, width, length, and spherical volumes, of endometriosis in the studied rat model (58, 59). Furthermore, Zhang et al. (60) applied rosiglitazone to surgically-induced endometriosis rats which enhanced the expression of PPARγ and impacted the development and progression of endometriosis by inhibition of angiogenesis as well as apoptosis induction. In 2002, Pritts et al. (61) described in their first relevant publication that rosiglitazone and 15 deoxy-Δ^12,14^ prostaglandin J_2_ decreases endometrial stromal cell transcription and translation of Regulated upon Activation Normal T-cell Expressed and Secreted (RANTES) *in vitro*. One year later, the same study group concluded that the addition of PPARγ ligands to endometriotic stromal cells inhibits RANTES promoter activity through a specific PPARγ response element (62). Moreover, Olivares et al. applied celecoxib in combination with rosiglitazone to a surgically induced endometriosis mouse model and outlined a statistically significant reduction in the mean number of lesions, the implant volume, or the vascularization level. Notably, the down-regulation of cell proliferation within the implants was statistically significant, while apoptosis showed a statistically significant boost (63). In 2010, Sharma et al. treated endometrial-endometriotic stromal cells isolated from ectopic endometrium with atorvastatin and observed a distinct expression increase of PPARγ (64). A year later, the same study group examined the therapeutic effect of rosiglitazone and 15d-PGJ2 on endometrial-endometriotic stromal cells and again reached the conclusion that both agents might support the expression of PPARγ (65).

Ohama et al. treated endometriotic stromal cells with pioglitazone and reported a significant reduction of the Tumor Necrosis Factor (TNF)-a-induced Interleukin-8 production, alongside a suppression of the growth of endometriotic stromal cells, as well as a reduction of the concentration of p65 (66). In addition, Kim et al. (67) treated infertile patients with stage III or IV endometriosis undergoing *in vitro* fertilization, alongside controlled ovarian stimulation with GnRH agonist, with pioglitazone and noticed both a significantly higher embryo implantation rate and significantly lower serum RANTES levels after pioglitazone treatment. In 2009, McKinnon et al. published their first original research article on the association of PPARγ and endometriosis. In peritoneal lesions, the pain reported by endometriosis patients increased as PPARγ expression augmented. Nonetheless, the extent of PPARγ expression did not correlate with the stage assigned to the patient (68). Three years later, the same study group treated endometrial stromal cells with ciglitazone and pioglitazone which attenuated a dose-dependent interleukin-6 and interleukin-8 release (69).

Wu et al. cultured immortalized endometrial and endometriotic cell lines with both the histone deacetylase inhibitor trichostatin A and ciglitazone and noted that trichostatin A up-regulates PPARγ expression in a dose-dependent way, while ciglitazone inhibits proliferation of endometriotic cells (70). Besides, Kavoussi et al. treated the endometrial epithelial cell line EM42 and the mesothelial cell line LP9 with the PPARγ agonist ciglitazone. Application of 40 mM ciglitazone to the EM42 cells decreased EM42 attachment to LP9 cells by 27%, while treatment of both EM42 and LP9 cells with 40 mM ciglitazone decreased EM42 attachment to LP9 by 37%. Administration of 40 mM ciglitazone to EM42 cells also decreased attachment to hyaluronic acid by 66%. Nevertheless, invasion of EM42 cells through the LP9 monolayer was not decreased by ciglitazone (71). In 2004, Lebovic et al. published their first original research article on the effects of ciglitazone in a rat model of endometriosis and reported that treatment with this thiazolidinedione might significantly decrease both the mean explant wet weight and the size of ectopic uterine tissues. Notably, the ciglitazone-treated rats experienced marked epithelial regression as well (72). Three years later, the same study group tested the effects of rosiglitazone in a baboon model of established endometriosis. The surface area of endometriotic lesions was statistically significantly higher in rosiglitazone non-treated baboons, with the rosiglitazone-treated baboons treated having a greater negative relative change in terms of peritoneal endometriotic lesion surface area (73). In 2010, the researchers conducted a prospective, randomized, placebo-controlled study in a baboon model to determine if, this time, pioglitazone could impede endometriosis development. Not only the surface area but also the volume of endometriotic lesions were significantly higher in the non-pioglitazone-treated baboons, with the overall number of endometriotic lesions being significantly greater (74). Three years later, Lebovic et al. revealed that ciglitazone succeeds in inhibiting the growth of the endometriotic epithelial cells 12Z up to 35% and of the endometriotic stromal cells 22B up to 70% via transformed cell cycle control and intrinsic apoptosis, diminishing the PGE2 receptor (EP) 2 and EP4 mRNA expression in 12Z and 22B cells, and down-regulating the expression and function of P450 aromatase mRNA and protein and estrone production in 12Z and 22B cells via EP2 and EP4 in a stromal-epithelial cell-specific way (75).

Wang et al. established a rat model of endometriosis by trans-implanting endometrial fragments to the peritoneal wall and then administered resveratrol to the rats. Intense staining of PPARγ expression was induced by high dose resveratrol treatment in glandular epithelial cells in the lesion tissues of model rats, hence inducing PPARγ activation in ectopic focus of endometriosis-induced rats (76).

In 2014, Nenicu et al. (77) proved that the angiotensin II type 1 receptor blocker telmisartan up-regulates PPARγ in peritoneal endometriosis-like lesions, hence resulting in a lower immune cell content, a reduced density of CD31-positive microvessels and a smaller number of Ki67-positive proliferating cells. Three years later, the researchers highlighted that co-treatment of peritoneal endometriotic lesions with telmisartan and the Cyclooxygenase (COX)-2 inhibitor parecoxib synergistically promotes the stromal and glandular expression of PPARγ (78).

Yang et al. established endometriosis rats with estradiol valerate and autologous transplantation and administered different doses of the Chinese traditional medicine QIU through oral gavage for 4 weeks, which promoted autophagy and inhibited angiogenesis by regulating the PPARγ/Nuclear factor kappa-light-chain-enhancer of activated B cells (NF-κB) signaling pathway (79).

**Figure 2:**
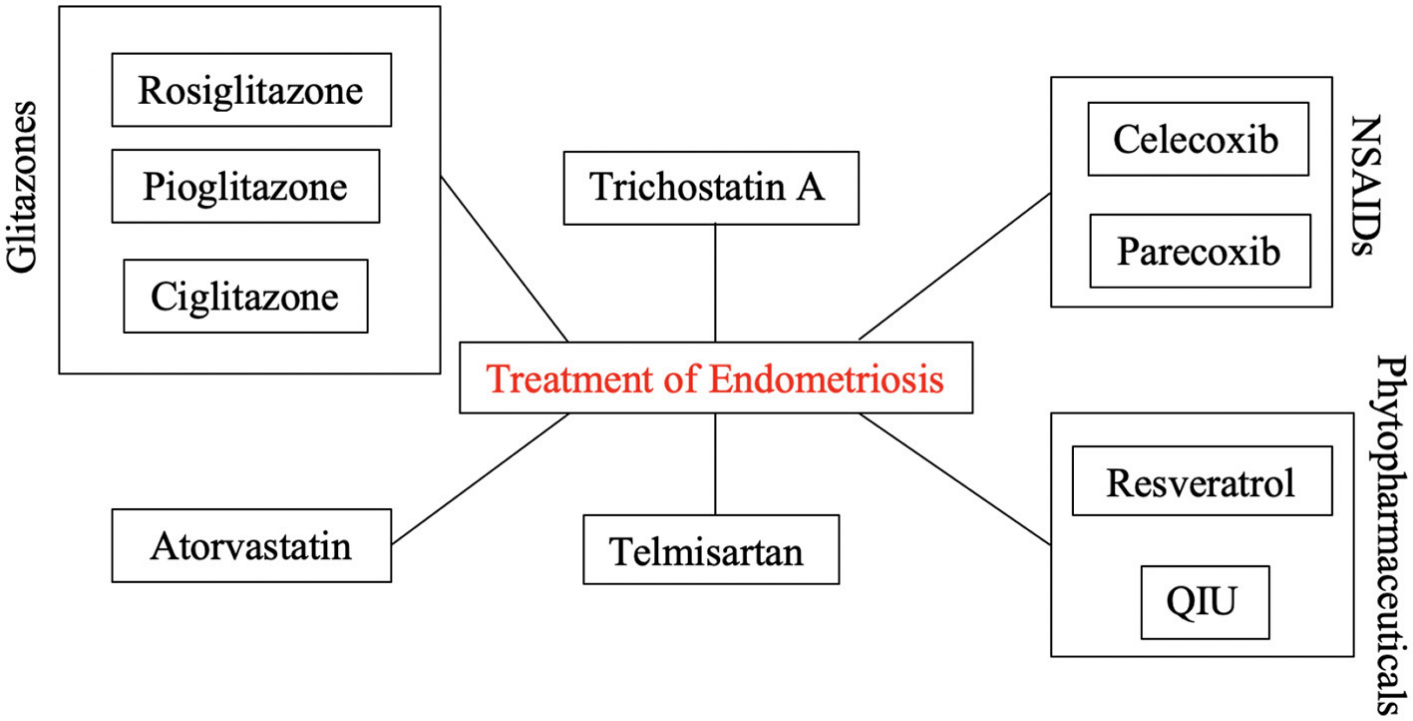
PPARγ-modulating treatment agents for the therapy of endometriosis. Selected well-established drug classes (analgesics; antidiabetics; etc.) seem to show promising therapeutic effects by up- or downregulating the expression of PPARγ in endometriotic cells.

Figure 2 depicts all identified PPARγ-modulating treatment agents that provenly play a significant role in the therapy of endometriosis.

## 4 Discussion

**Figure 3:**
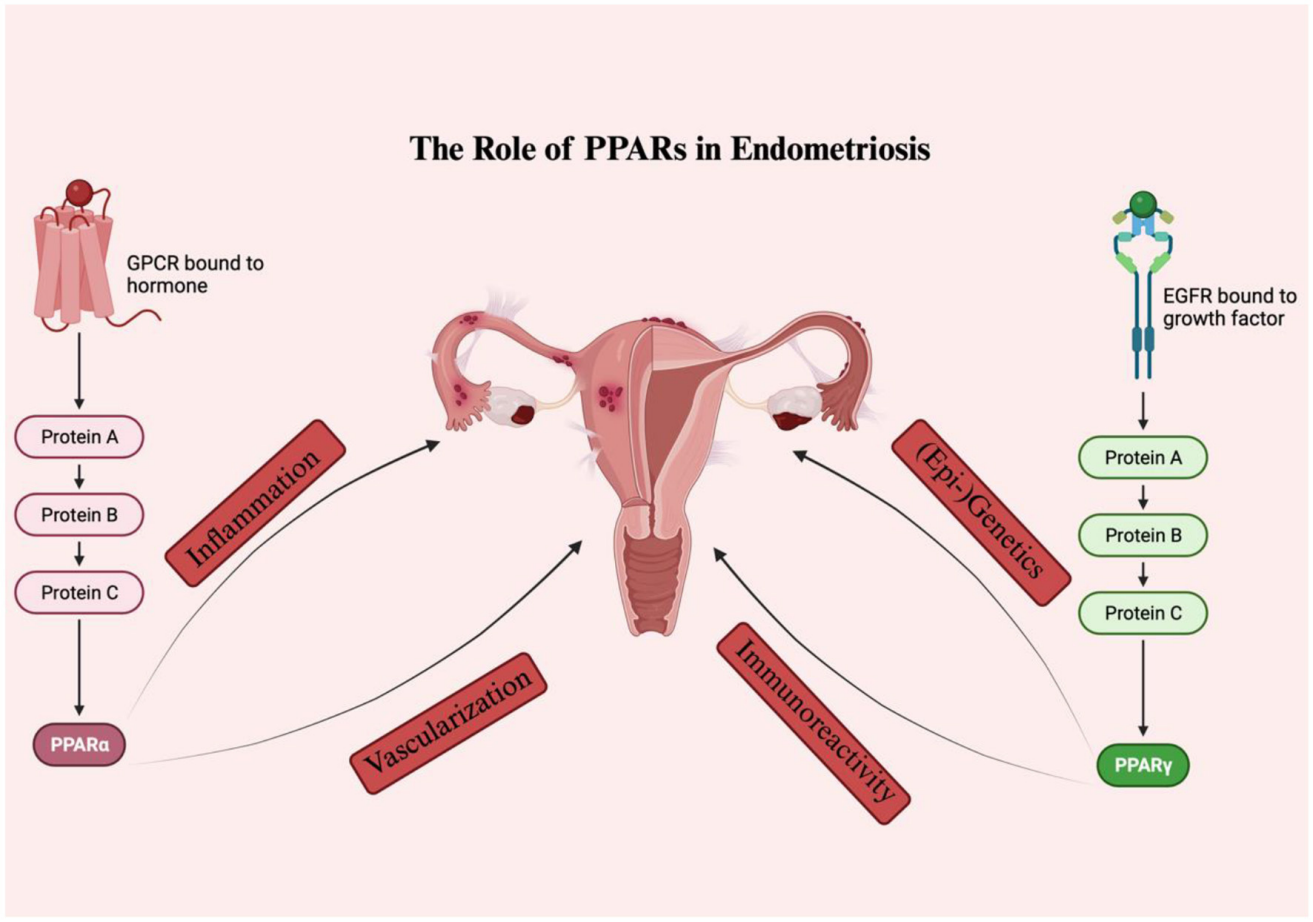
The role of PPARS in endometriosis. PPARα and PPARγ seem to influence various pathways involved in the disease onset and progression, including inflammation, vascularization, immunoreactivity, as well as (epi-) genetics.

It has been shown that all PPAR isoforms are expressed in the uteri of various mammal species (80). However, the physiological status of each person or species may be the cause of the variation in their expression patterns (80). PPARs appear to be involved in the regulation of numerous uterine secretory functions essential for the implantation of the embryo during pregnancy or the lysis of the corpus luteum during the estrous cycle (81–83) given their differential expression during different reproductive conditions. Their interactions with prostaglandins, steroids, and cytokines may act as a conduit for their effects at the endometrial level (80). Additionally, alterations in the PPAR expression profile appear to have an effect on signal transduction during inflammatory processes that occur in the endometrium in cases of cystic endometrial hyperplasia and pyometra, as well as to cause hormonal disturbances (84). It is interesting to note that PPAR agonists have been shown to be correlated with estradiol-induced hyperplasia and proliferation in mouse uterus (85). So far, only few drugs such as hormonal therapy with progesterone or GnRH-modulators have received FDA approval for the treatment of endometriosis (86). Up to the present time, no review article has, to our knowledge, been published focusing on the role of PPARs in endometriosis. As such, the current article constitutes an up-to-date review comprehensive of the literature about the effects of the PPAR isoforms PPARα and PPARγ in endometriosis establishment and progression, alongside their potential as eventual anti-endometriotic treatment targets (Figure 3).

Only two study groups have, so far, investigated the role of PPARα in endometriosis. Based on the study results, negative regulation of the PPARα pathway seems to promote endometriosis progression and to hinder adequate vascularization which directly correlates with the level of inflammation at the endometriotic implantation site. In the context of PPARα therapeutic targeting, the administration of resveratrol in endometriosis seemingly leads to the activation of PPARα which might potentially contribute to the recovery from this disease. Aspirin and other NSAIDs also succeed in inducing the PPARα expression.

PPARγ undoubtedly represents the most studied PPARγ isoform in endometriosis. Patients with endometriosis tend to have altered levels of PPARγ, with both ovarian endometriomas and deep infiltrating endometriosis showing significantly reduced immunoreactivity against PPARγ, but PPARγ activity being higher in abdominal wall endometriosis than in the eutopic endometrium. Interestingly enough, the exact location and extent of the endometriosis seems to have an influence on the PPARγ expression pattern. Additionally, the PPAR-γ2 Pro12Ala polymorphisms seem to differentially correlate with the risk of endometriosis in different ethnic populations. For instance, no association between distribution of genotype or allele frequencies for the PPAR-γ Pro12Ala polymorphism and the presence of adenomyosis and/or endometriosis could be noticed in the Japanese population. In the Korean population, on the other hand, the distribution of the PPAR-γ2 Pro12Ala polymorphism differs between advanced-stage endometriosis patients and healthy women. Significantly, genetic variants of the PGC-1α do not seem to correlate with the endometriosis risk in Indian female patients.

In the context of PPARγ therapeutic targeting, glitazones such as rosiglitazone, pioglitazone, and ciglitazone, seem to embody potent treatment agents which successfully suppress inflammation in endometriosis, decrease both the mean explant wet weight and the size of ectopic uterine tissues and even endorse fertility. Resveratrol treatment induces PPARγ activation in ectopic focus of endometriosis, while telmisartan up-regulates PPARγ in peritoneal endometriosis-like lesions with consecutive anti-inflammatory and anti-proliferative effects. Impressively, the Chinese traditional medicine QIU seems to effectively induce autophagy and block angiogenesis via the PPARγ/NF-κB signaling pathway.

Unfortunately, no study has been found that has, up to this point, included a sizable number of tissue samples, which would have provided more reliable and objective results. Furthermore, no PPAR agonist was given to patients with endometriosis as part of randomized controlled trials in order to assess the clinical applicability/efficacy of these drugs and identify potential unknown toxic effects. Future research should therefore address the aforementioned drawbacks and, ideally, examine the function of PPARs in early and advanced stage endometriosis separately. Most importantly, the efficacy of these novel agents on specific symptoms of endometriosis, namely infertility or chronic pelvic pain, should be thoroughly tested, given that these are two very different outcomes and therapeutic targets for various drugs.

The nonsystematic methodology used in the study selection is one of the review's limitations. In spite of the fact that systematic literature reviews adhere to strict rules and standards and represent the most accurate method for locating pertinent research articles, this methodology necessitates a precise research question by excluding broader topics like the function of PPARs in endometriosis. The publication bias (as data from statistically insignificant studies are less likely to be published), and the associated evidence selection bias, are also drawbacks of the present study.

## 5 Conclusions

In conclusion, the present literature review emphasizes the critical role of PPARs in the onset and progression of endometriosis and emphasizes the therapeutic potential of PPAR agonists against endometriotic cells. In order to come to unbiased and repeatable results and fully delineate the role of PPARs in endometriosis pathogenesis and therapy, additional systematic research is still needed in this field including knockout or knockdown study models and implementation of up- or down-regulators.

## Author contributions

IP: Conceptualization, Methodology, Writing—original draft. ST: Writing—review & editing. MB: Writing—review & editing.
